# Upregulation of long non-coding RNA HOXA-AS2 promotes proliferation and induces epithelial-mesenchymal transition in gallbladder carcinoma

**DOI:** 10.18632/oncotarget.16561

**Published:** 2017-03-25

**Authors:** Peng Zhang, Peihua Cao, Xiaofeng Zhu, Mingxin Pan, Kebo Zhong, Rui He, Yang Li, Xingyuan Jiao, Yi Gao

**Affiliations:** ^1^ Department of Hepatobiliary Surgery II, Zhujiang Hospital, Southern Medical University, Guangzhou 510280, Guangdong, P. R. China; ^2^ Organ Transplant Center, The First Affiliated Hospital, Sun Yat-Sen University, Guangzhou 510080, Guangdong, P. R. China; ^3^ Guangdong Provincial Research Center of Artificial Organ and Tissue Engineering, Zhujiang Hospital, Southern Medical University, Guangzhou 510280, Guangdong, P. R. China; ^4^ State Key Laboratory of Organ Failure Research, Southern Medical University, Guangzhou 510515, Guangdong, P. R. China

**Keywords:** HOXA-AS2, LncRNA, GBC, metastasis, EMT

## Abstract

Gallbladder carcinoma (GBC) is the most common malignancy of the bile duct and patients with GBC have extremely poor prognoses. Increasing evidence indicates that long non-coding RNAs (lncRNAs) regulate diverse cellular processes, including cell growth, differentiation, apoptosis, and cancer progression. However, the function of lncRNAs in the progression of GBC remains largely unknown. Here, we reported that HOXA cluster antisense RNA2 (HOXA-AS2) was upregulated in GBC. *In vitro* experiments revealed that HOXA-AS2 knockdown significantly inhibited GBC cells proliferation by causing G1 arrest and promoting apoptosis, whereas HOXA-AS2 overexpression promoted cell growth. Further functional assays indicated that HOXA-AS2 overexpression significantly promoted GBC cell migration and invasion by promoting EMT. Taken together, our study demonstrates that HOXA-AS2 could act as a functional oncogene in GBC, as well as a potential therapeutic target to inhibit GBC metastasis.

## INTRODUCTION

Gallbladder carcinoma (GBC) is a malignant cancer originating from the biliary tract. In recent years, the incidence and prevalence of GBC are increasing in the developed world [1–2]. In the locally advanced setting, they are treated with curative resection [3–4]. GBC is most often diagnosed at an advanced stage with lymph-node metastases because the early stages of GBC progression are largely asymptomatic [5]. A significant number of these patients ultimately die from metastatic disease [6]. Therefore, to clearly elucidate the mechanism of GBC biology and to design effective therapeutic strategies are very important for patients with GBC.

Long noncoding RNA (lncRNA) is a type of noncoding RNA greater than 200 nucleotides in length [7]. Recent studies showed that lncRNA can function as an oncogene or tumor suppressor in various malignant tumors [8]. Abnormal lncRNA expression has been implicated in the regulation of physiological and pathological processes of GBC, indicating that lncRNAs could be utilized for GBC diagnosis and prognosis [9–10]. HOXA cluster antisense RNA 2 (HOXA-AS2), a lincRNA located between and antisense to the human HOXA3 and HOXA4 genes [11]. Recently evidence has shown that HOXA-AS2 promote several tumorigenic features including survival, proliferation and invasion. However, the biological functions and significance of HOXA-AS2 in GBC has not yet been established. In this study, we found that HOXA-AS2 expression was upregulated in GBC tissues, and upregulation of HOXA-AS2 was also correlated with larger tumor size and advanced pathologic stage. Moreover, lncRNA HOXA-AS2 promotes GBC cell growth and invasion *in vitro*. These results suggest that HOXA-AS2 may be a potential biomarker for GBC diagnosis and gene therapy.

## RESULTS

### Differential expression of HOXA-AS2 in GBC tissues and cell lines

**Figure 1:**
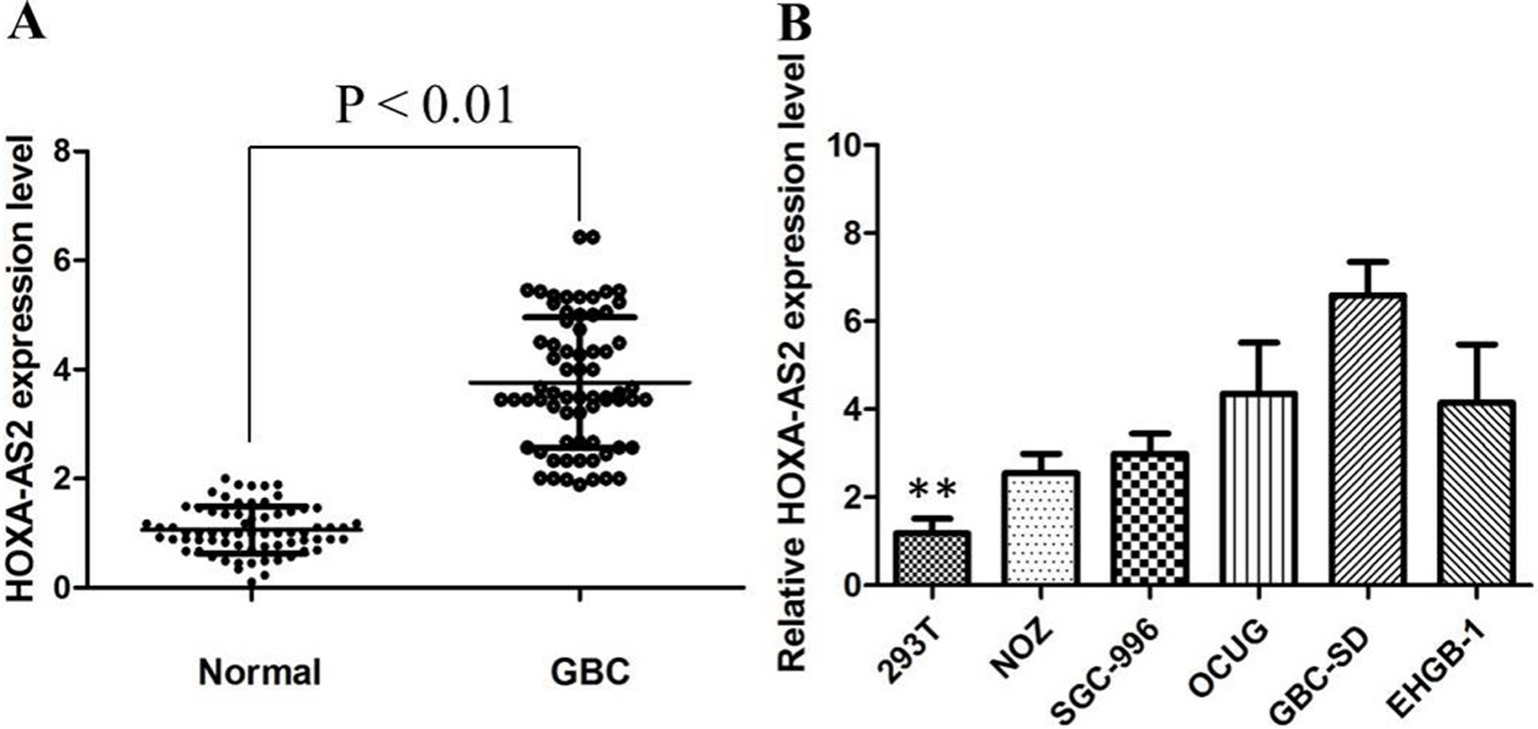
(**A**) HOXA-AS2 was detected in GBC tissues and adjacent noncancerous tissues by qRT-PCR; (**B**) qRT-PCR showing expression level of HOXA-AS2 in GBC cell lines.

HOXA-AS2 expression in primary GBC tissues was evaluated by qRT-PCR and normalized to an internal control. Expression of HOXA-AS2 in GBC tissues is significantly higher than that of in normal paired tissues (Figure 1A). Noticeably, high HOXA-AS2 expression in GBC was significantly correlated with GBC tumor size (*P* < 0.01), tumor stage (T stage) (*P* < 0.01), and node stage (N stage) (*P* < 0.01). These data suggested that high level of HOXA-AS2 expression was associated with GBC progression.

**Figure 2:**
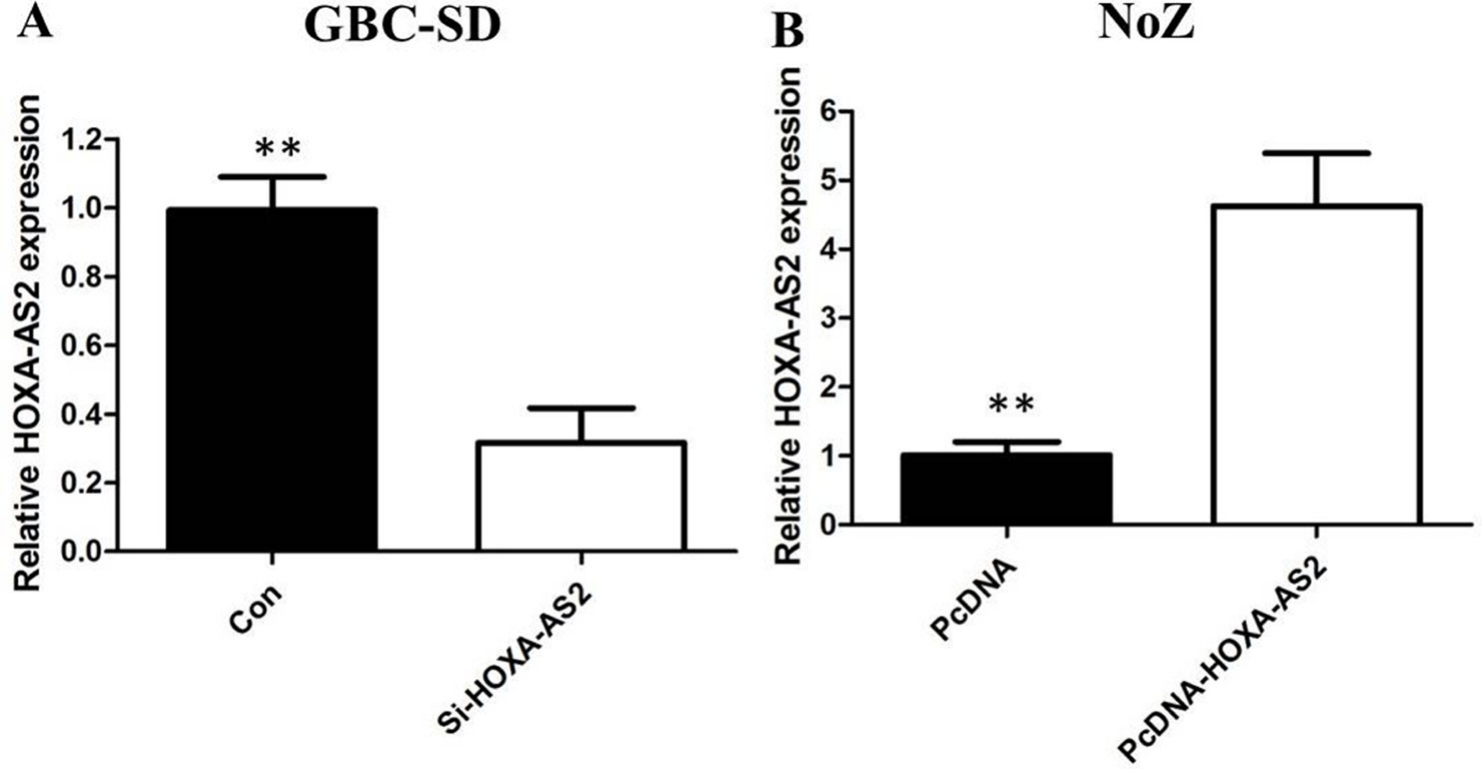
(**A**) The qRT-PCR assay revealed that HOXA-AS2 was efficiently downexpression by transfected with siRNA in GBC-SD cells; (**B**) The qRT-PCR assay revealed that HOXA-AS2 was efficiently overexpression in NoZ cells transfected with pCDNA-HOXA-AS2.

To investigate the functional role of HOXA-AS2 in GBC cells, the qRT-PCR was performed to detect the expression of HOXA-AS2 in diverse GBC cell lines. As shown in Figure 1B, the expression of SPRY4-IT1 was observed to be higher in all five GBC cell lines compared with the 293T cell line. Among all GBC cell lines, GBC-SD cells showed higher expression of SPRY4-IT; however, NoZ cells showed lower expression of SPRY4-IT1. Thus, we used NoZ and GBC-SD cells as a model to investigate the biological consequences of HOXA-AS2 in regulating cancer cell proliferation and invasion. Then, HOXA-AS2 siRNA was transfected in to GBC-SD cell lines, which have the highest levels of HOXA-AS2. Conversely, for gain of function studies, a pcDNA-HOXA-AS2 vector was transiently transfected to ectopically overexpress SPRY4-IT1 in the NoZ cell line. Successful RNAi-mediated knockdown and ectopic expression of HOXA-AS2 in NoZ and GBC-SD cells respectively, were confirmed by qRT-PCR on harvested RNA 48 hours after transfection (Figure 2A and 2B).

### HOXA-AS2 promotes GBC cells proliferation *in vitro*

**Figure 3:**
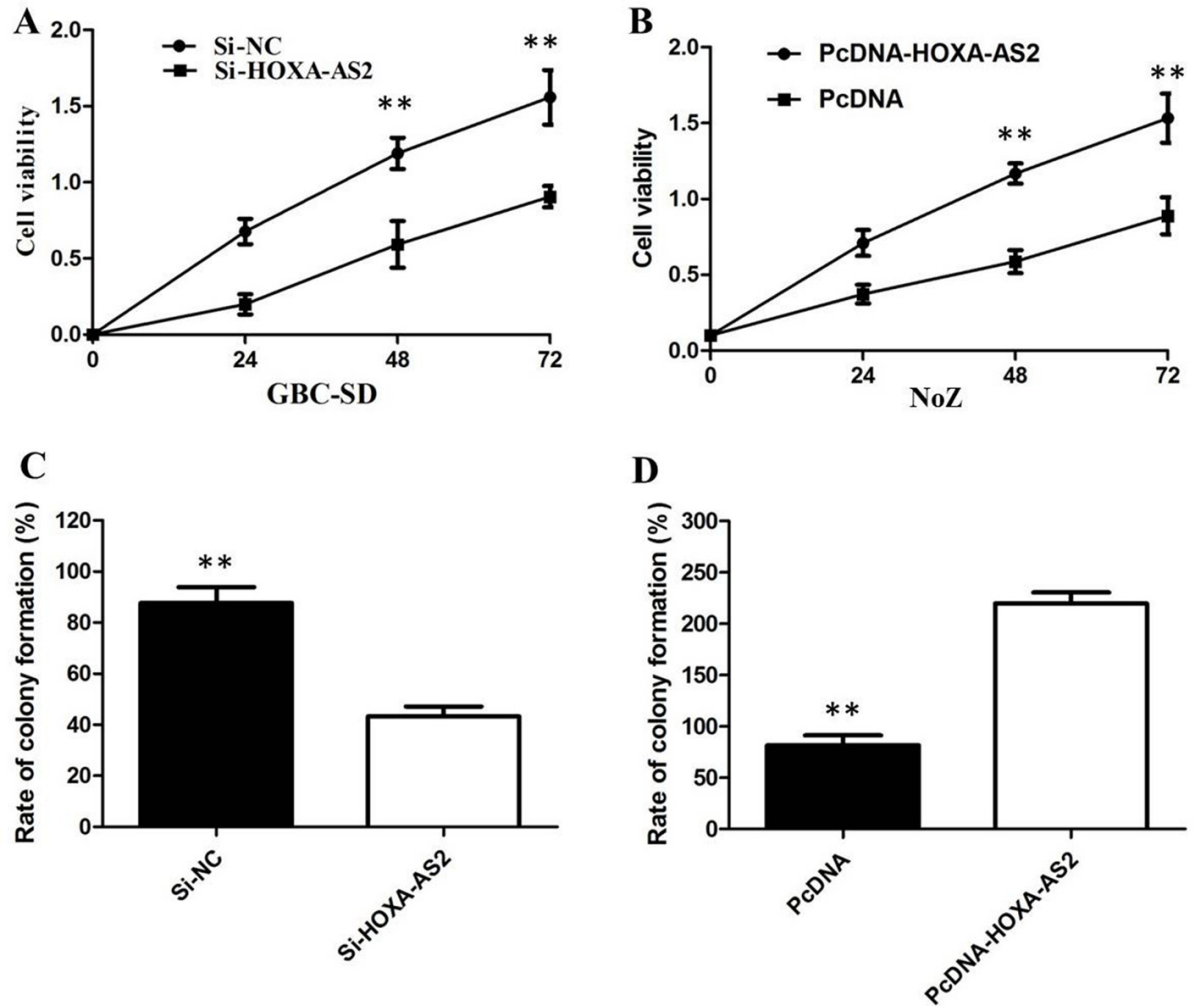
(**A**) CCK8 assay showing knockdown of HOXA-AS2 inhibited cell proliferation of GBC-SD cells; (**B**) CCK8 assay showing overexpression of HOXA-AS2 promoted cell proliferation of NoZ cells; (**C**) Colony-formation assays showed that silencing of HOXA-AS2 significantly inhibited the colony-forming ability of GBC-SD cells; (**D**) Colony-formation assays showed that overexpression of HOXA-AS2 significantly promoted the colony-forming ability of NoZ cells.

CCK8 and colony-formation assay showed that knockdown of HOXA-AS2 expression significantly inhibited cell proliferation in GBC-SD cell lines compared with the control cells (Figure 3A, 3C). Our results showed that the growth of NoZ cells transfected with pCDNA-HOXA-AS2 was increased compared with control cells (*P* < 0.05; Figure 3B). Colony formation assay results revealed that clonogenic survival was incresaed following overexpression of HOXA-AS2 in NoZ cells (*P* < 0.05; Figure 3D).

### Knockdown of HOXA-AS2 induced GBC cells apoptosis

**Figure 4:**
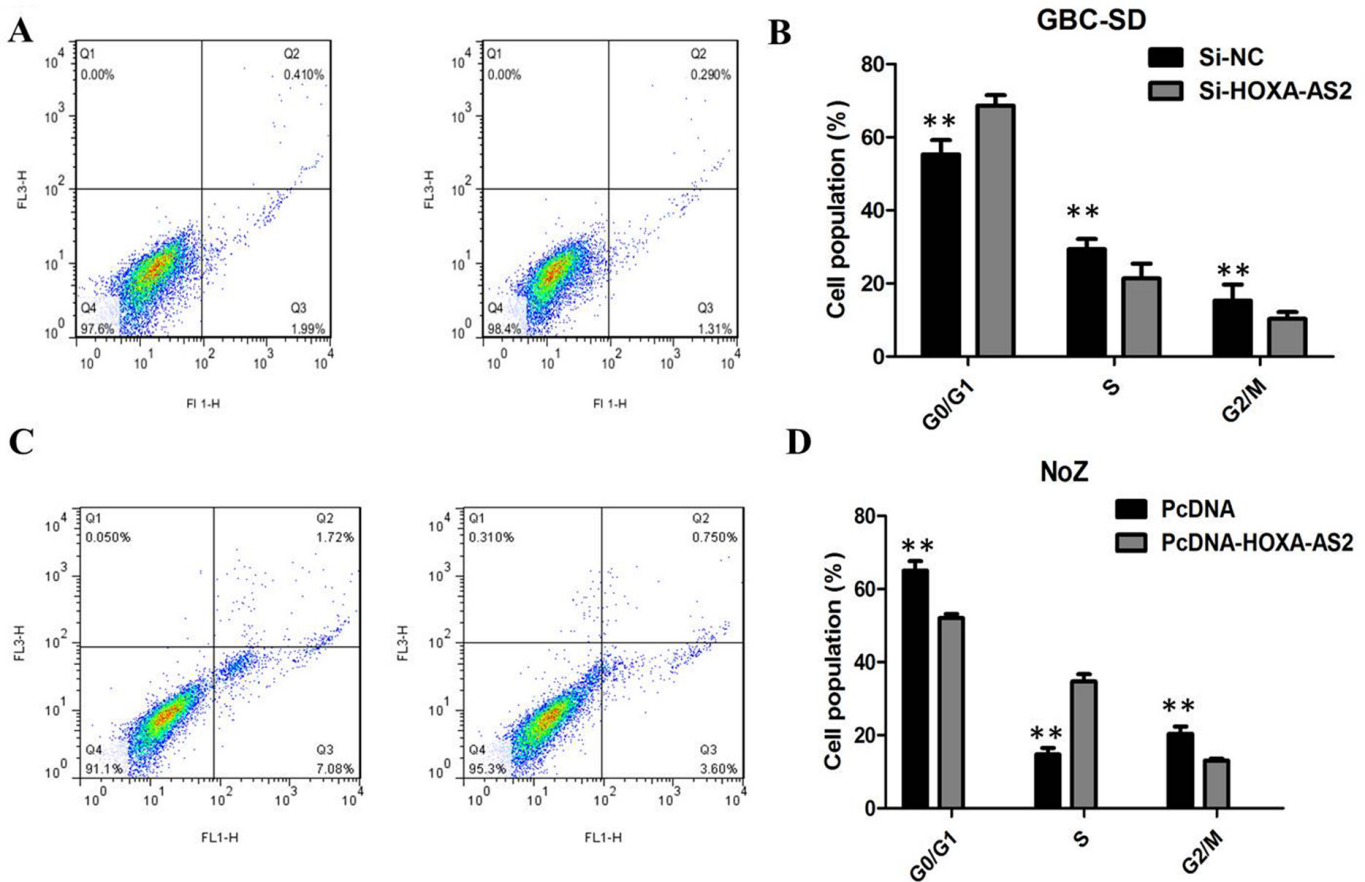
(**A**) Knockdown of HOXA-AS2 resulted in an increase of apoptotic rate of GBC-SD cells; (**B**) Overexpression of HOXA-AS2 resulted in an decrease of apoptotic rate of NoZ cells; (**C**) Knockdown of HOXA-AS2 resulted in cell arrest in G1 phase of GBC-SD cells; (**D**) Overexpression of HOXA-AS2 increased the S-phase pencentage and decreased G0/G1 phase percentage of NoZ cells.

We preformed flow cytometry to determine whether the effect of HOXA-AS2 on GBC cells proliferation reflected cell apoptosis. The results showed that GBC-SD cells transfected with HOXA-AS2 siRNA had higher apoptotic rate in comparison with control cells (Figure 4A). Next, flow cytometric analysis revealed that knockdown of HOXA-AS2 resulted in cell arrest in G1 phase of cell cycle in GBC cell lines (Figure 4C). As expected, the cell apoptosis was markedly decreased by pcDNA-HOXA-AS2 (Figure 4B). Overexpression of HOXA-AS2 increased the S-phase percentage and decreased G0/G1 phase percentage of NoZ cells (Figure 4D). These data indicate that HOXA-AS2 could promote the proliferation phenotype of GBC cells by altering the apoptosis or cell cycle progression.

### HOXA-AS2 promotes the migration and invasion of GBC cells via regulating EMT

**Figure 5:**
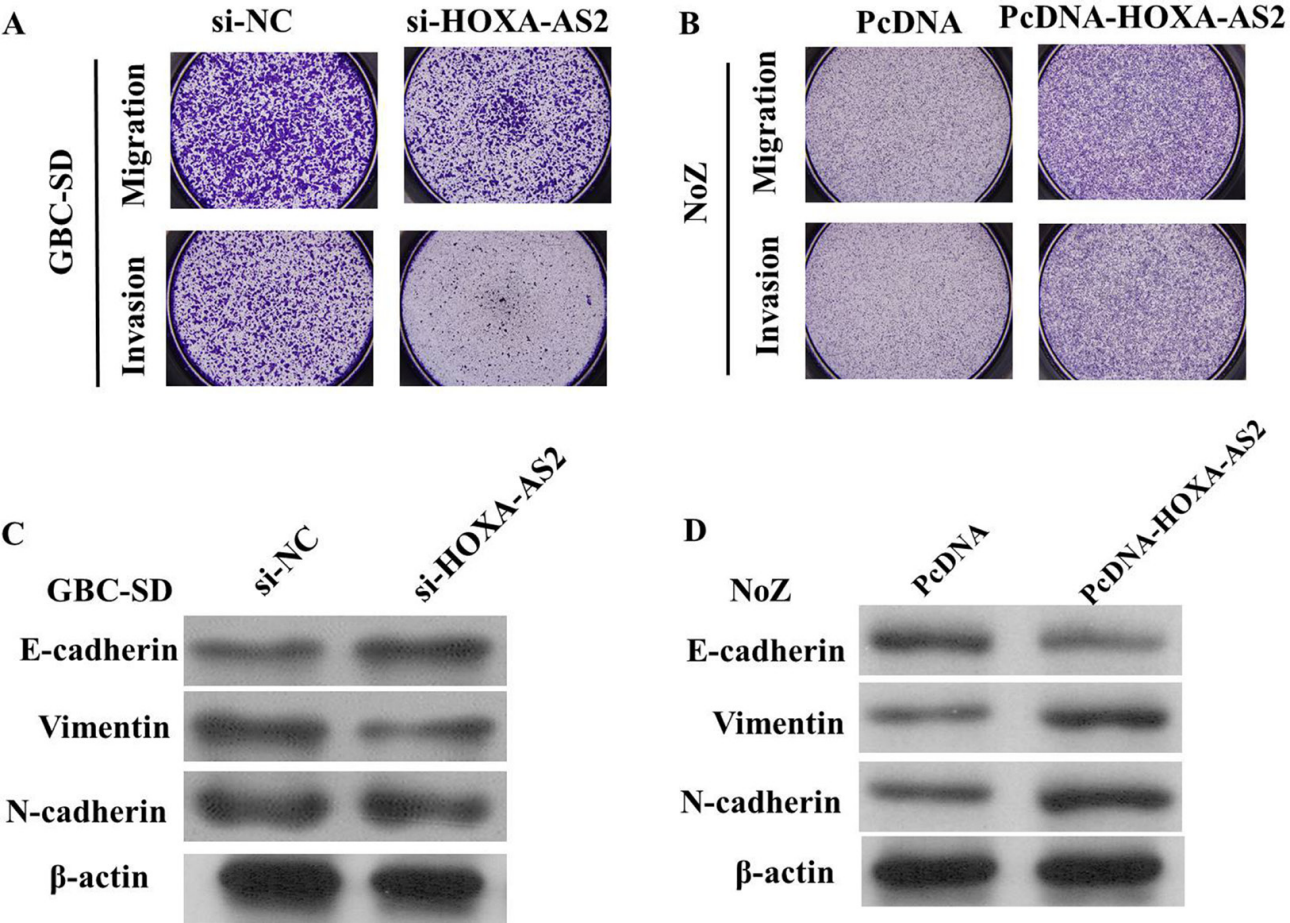
(**A**) The migration and invasion of GBC-SD cell line were significantly inhibited by si-HOXA-AS2; (**B**) The migration and invasion of NoZ cell line were significantly increased by si-HOXA-AS2; (**C**) Knockdown of HOXA-AS2 reversed EMT in GBC-SD cells; (**D**): Overexpression of HOXA-AS2 promoted EMT in NoZ cells

We perform transwell assay to investigate the effects of HOXA-AS2 on migration and invasion of GBC cell lines. As shown in Figure 5A, the migration and invasion of GBC-SD cell line were significantly inhibited by si-HOXA-AS2. The migration and invasion activity of HOXA-AS2-overexpressing cells was significantly increased in NoZ cells (*P* < 0.05; Figure 5B).

Because EMT is vital for cell invasion, we next examined whether silencing HOXA-AS2 expression inhibited mesenchymal features. As expected, HOXA-AS2 knockdown decreased the expression of Vimentin and N-cadherin, and increased E-cadherin expression in GBC-SD cells (Figure 5C and 5D), while, overexpression of HOXA-AS2 could decrease E-cadherin and increase Vimentin expression in NoZ cells, suggeating that inhibition of HOXA-AS2 in GBC cells changed the cell morphology from a mesenchymal to a more epithelial phenotype.

## DISCUSSION

GBC is the most common malignancy of the bile duct. Due to non-specific symptoms in the early stage and no effective screening techniques, the majority of GBC patients are diagnosed at advanced stages, resulting in a poor prognosis [12–13]. Thus, research on GBC early detection and improvement of current treatment strategies is urgent.

The long non-coding RNA with a length of > 200 nt gained substantial attention recently [14]. An increasing number of studies have shown that lncRNAs may play a fundamental role in a variety of biological cellular processes and multiple diseases, including cancer [15]. Numerous new lncRNA molecules have been proved to be involved in the tumorigenesis and progression of GBC. LncRNA-MALAT-1 was abnormally upregulated in GBC and played an oncogenic role in GBC cells [9]. Recently, HOXA-AS2, a long non-coding RNA located between the HOXA3 and HOXA4 genes in the HOXA cluster, has been characterized as an oncogene in various cancers, including acute promyelocytic leukemia and gastric cancer. Inhibition of HOXA-AS2 could suppress cell proliferation and induce cell apoptosis [11]. However, whether HOXA-AS2 is involved in the progression of HCC is ill-defined and the molecular mechanisms remain to be fully elucidated. In the present study, we found that HOXA-AS2 mRNA was overexpressed in GBC tumor tissues and HOXA-AS2 expression was associated with GBC progression. The analysis of tumor cell proliferation and invasion abilities in lncRNA HOXA-AS2-knockdowned cells showed the inhibitory role of lncRNA HOXA-AS2 in GBC, which is similar to its role in promyelocytic leukemia and gastric cancer. We further identified that expression of EMT related markers were significantly altered following HOXA-AS2 knockdown. Thus, our data suggests that overexpression of HOXA-AS2 might promote ovarian cancer progression by regulating EMT.

The invasion and metastasis of cancer cells are landmark events that involve many changes in cellular behavior, and lead to different steps of the metastatic cascade [16]. Although HOXA-AS2 can suppress migratory and invasive phenotype of various cancer cells, the underlying mechanism is still elusive. Our results showed that inhibition of HOXA-AS2 impairs cell invasion and metastasis through the regulation of EMT process. EMT is a biological process where epithelial cells lose their polarity and undergo transition into a mesenchymal phenotype. To investigate the affect of lncRNA HOXA-AS2 on the epithelial-mesenchymal transition of GBC cells, the study examined the mesenchymal markers and epithelial markers in GBC cells with or without lncRNA HOXA-AS2 overexpression. It showed that E-cadherin (epithelial marker) was downregulated while Vimentin and N-cadherin (mesenchymal markers) were upregulated in lncRNA HOXA-AS2 overexpressed cells, indicating that effects of HOXA-AS2 on cell migration and invasion were partly associated with EMT process.

In conclusion, our study showed that HOXA-AS2 expression level was obviously elevated in GBC tissues and cells lines and corrected with the malignant status in GBC patients. Furthermore, knocking down HOXA-AS2 expression significantly inhibited GBC migration and invasion *in vitro* and regulated EMT-associated proteins expression.

## MATERIALS AND METHODS

### Patients and samples

Sixty-eight paired GBC tissue samples and neighboring noncancerous gallbladder tissues were obtained from patients who underwent surgery at Department of Hepatobiliary Surgery, Zhujiang Hospital, Southern Medical University between 2008 and 2015. All patients were staged according to the tumor node metastasis (TNM) staging system (the 7th edition) of the American Joint Committee on Cancer (AJCC) staging system. These tissue specimens were snap-frozen in liquid nitrogen and stored at −80°C until use. This study was approved by institutional review board, and informed consent papers were obtained from all of the patients.

### Cell culture

The human GBC cell lines NOZ, SGC-996, OCUG, GBC-SD and EHGB-1 cell lines, and the 293T cell line were purchased from the Institute of Biochemistry and Cell Biology of the Chinese Academy of Sciences (Shanghai, China). The cell lines were cultured in Dulbecco's modified Eagle's medium (Gibco BRL, Grand Island, NY, USA), supplemented with 10% fetal bovine serum (FBS, HyClone, Invitrogen, Camarillo, CA, USA), and 100 ug/ ml penicillin and 100 μg/ml streptomycin (Invitrogen, Carlsbad, CA, USA). Cells were incubated at 37°C with 5% CO_2_.

### RNA extraction and qRT-PCR assays

Total RNA was extracted from tissues or cultured cells using TRIZOL reagent (Invitrogen). For qRT-PCR, RNA was reverse transcribed to cDNA by using a Reverse Transcription Kit (Takara, Dalian, China). Real-time PCR analyses were performed with SYBR Premix Ex Taq (Takara, Dalian China). Results were normalized to the expression of GAPDH. The sequence of the primers were as following: HOXA-AS2 (Forward: 5′-CCCGTAGGAAGAACCGATGA-3′, Reverse: 5′-TTTAGGCCTTCGCAGACAGC-3′) and GAPDH (Forward: 5′-GGGAGCCAAAAGGGTCAT-3′, Reverse: 5′-GAGTCCTTCCACGATACCAA-3′). The qRT-PCR assays were conducted on an ABI 7500, and data collected with this instrument. Our qRT-PCR results were analyzed and expressed relative to threshold cycle (CT) values, and then converted to fold changes.

### Transfection

The small interfering RNA-HOXA-AS2 (si-HOXA-AS2) or pcDNA- HOXA-AS2 were transfected into cells using Lipofectamine^®^ 2000 Reagent (Thermo Fisher Scientific, USA) in a 6-well cell culture plate following to the manufacturer's instructions. Plasmid vectors (sh-HOXA-AS2 and empty vector) for transfection were extracted by DNA Midiprep kit (Qiagen, Hilden, Germany). The full-length complementary DNA of HOXA-AS2 was synthesized by Realgene (Nanjing, China) and subcloned into the pcDNA3.1 (+) vector (Invitrogen) according to the manufacturer's instructions. At 48 h post-transfection, cells were harvested for qRT-PCR or western blot analysis.

### CCK8 assay

The cell proliferation was measured using the WST-8 kit (YiSheng, ShangHai, China). The transfected cells were plated into 96-well plates (Corning Costar, Corning, NY) at a density of 1.0 × 103/well/100 μL, and then 10 μL of CCK8 solution was added to each well, followed by incubation for 2 h. The cell viability was determined by measuring the absorbance at 450 nm. Measurement of cell proliferation was done once every.

### Colony formation assay

Cell transfection was performed 48 h later as above mentioned. The transfected cells were harvested after trypsinization, counted and plated at a density of 500 cells/6-cm dish. The medium was refreshed once every 3 days. Ten days later, cells were washed in 1 × PBS twice, fixed in 3.7% methanol, stained with 0.1% crystal violet and then counted. The cell colony was counted (each colony contains at least 50 cells).

### Flow cytometric analysis

Cells were harvested directly or 48 h after siRNA transient transfection and washed with ice-cold phosphate-buffered saline (PBS). The PI/RNase staining kits (Multisciences, Hangzhou, China) and annexin V-fluorescein isothiocyanate (FITC) apoptosis detection kits (KeyGEN Biotech, Nanjing, China) were used to detect cell cycle and apoptosis in a FACScan instrument (Becton Dickinson,, Mountain View, CA, USA), respectively.

### Transwell migration/invasion assay

Transwell chamber was used to measure cell migration and invasion abilities. In brief, culture inserts with 8-mm pore size (Transwell; Corning, NY) were placed into 24-well plates. Before the measurement of invasion ability, the plates were pre-coated with matrigel. 2 h before the addition of matrigel, 500 μL of serum-free medium was independently added to the upper and lower chambers, followed by incubation at 37°C for hydration. Cells were digested by typsin, and resuspended in serum-free medium. The cell density was adjusted to 1 × 105/mL. Then, 200 μL of cell suspension was added into the upper chamber, and 500 μL of DMEM containing 10% FBS into the lower chamber. After incubation at 37°C with 5% CO2 for 24 h, the Transwell chamber was removed, cells were washed with 1 × PBS, fixed in paraformaldehyde for 20 min, and then stained with 0.1% crystal violet for 20 min. The cotton swab was used to clean the non-migrated cells in the upper chamber, cells migrating through the membrane were counted in 5 randomly selected fields under a microscope (Nikon) at a magnification of ×100.

### Western blot analysis

The indicated cells were washed twice with precold phosphate-buffered saline quickly and suspended in extraction buffer. Proteins were quantied by Bradford method. Then, 50mg of total protein extracts was fractionated by 10% sodium dodecyl sulfate-polyacrylamide gel electrophoresis and transferred to polyvinylidene diuoride membranes (GE Healthcare, Piscataway, NJ, USA). The membranes were blocked with 5% milk at room temperature for 2 h. The primary antibodies including anti E-cadherin, anti-N-cadherin, anti-Vimentin (Santa Cruz Bio-technology, Santa Cruz, CA, USA), and anti-β-actin antibody (Cell Signaling Technology) were added and incubated with blots at 4°C for 12 h. Membranes were then washed three times with TBST and incubated with the corresponding secondary antibodies conjugated to horseradish peroxidase for 1 h at room temperature. Membranes were then washed again three times for 10 min each with TBS-T. Target protein bands were visualized using the enhanced chemiluminescence method. The intensity of the bands was quantified using the Tanon GIS syatem (Tanon, Shanghai, China) and the data were normalized to β-actin loading controls. All western immunoblot analyses were performed three times.

### Statistical analysis

Data were presented as mean ± SEM. Group comparison was performed by Student's *t*-test. *P* value < 0.05 was considered as significant difference. *, **, and *** donates significance at 0.05, 0.01 and 0.001 level respectively.
